# ^1^H-NMR metabolomic profile of healthy and osteoarthritic canine synovial fluid before and after UC-II supplementation

**DOI:** 10.1038/s41598-022-23977-1

**Published:** 2022-11-16

**Authors:** Marzia Stabile, Chiara Roberta Girelli, Luca Lacitignola, Rossella Samarelli, Antonio Crovace, Francesco Paolo Fanizzi, Francesco Staffieri

**Affiliations:** 1 Section of Veterinary Clinics and Animal Production, Department of Emergency and Organ Transplantation, University of Bari, 70123 Bari, Italy; 2 Department of Biological and Environmental Sciences and Technologies, University of Salento, 73100 Lecce, Italy; 3 Section of Avian Pathology, Department of Veterinary Medicine, University of Bari, 70123 Bari, Italy

**Keywords:** Musculoskeletal system, Zoology, Metabolomics

## Abstract

The aim of the study was to compare the metabolomic synovial fluid (SF) profile of dogs affected by spontaneous osteoarthritis (OA) and supplemented with undenatured type II collagen (UC-II), with that of healthy control dogs. Client-owned dogs were enrolled in the study and randomized in two different groups, based on the presence/absence of OA (OA group and OA-free group). All dogs were clinically evaluated and underwent SF sampling for ^1^H-Nuclear Magnetic Resonance spectroscopy (^1^H-NMR) analysis at time of presentation. All dogs included in OA group were supplemented with UC-II orally administered for 30 days. After this period, they were reassessed (OA-T30). The differences in the ^1^H-NMR metabolic SFs profiles between groups (OA-free, OA-T0 and OA-T30) were studied. The multivariate statistical analysis performed on SFs under different conditions (OA-T0 vs OA-T30 SFs; OA-T0 vs OA-free SFs and OA-T30 vs OA-free SFs) gave models with excellent goodness of fit and predictive parameters, revealed by a marked separation between groups. β-Hydroxybutyrate was identified as a characteristic compound of osteoarthritic joints, showing the important role of fat metabolism during OA. The absence of β-hydroxybutyrate after UC-II supplementation suggests the supplement’s effectiveness in rebalancing the metabolism inside the joint. The unexpectedly high level of lactate in the OA-free group suggests that lactate could not be considered a good marker for OA. These results prove that ^1^H-NMR-based metabolomic analysis is a valid tool to study and monitor OA and that UC-II improves clinical symptoms and the SF metabolic profile in OA dogs.

## Introduction

Osteoarthritis (OA) is a widely recognized chronic illness that affects the entire joint. This pathological condition causes significant pain and disability and negatively impacts the well-being of both humans and dogs^1–3^. The development and progression of OA are similar in both species^4^ and are influenced by multiple variables, including the immune system, local tissue injuries and metabolic dysfunction^4–7^.

It was estimated that 20% of the canine population over 1 year of age is affected by OA^8,9^. A cure for OA is still unknown, but impressive efforts have been made by scientific researchers for the development of novel strategies for the diagnosis and management of pain and joint dysfunction^1,10,11^.

In the field of OA, undenatured type II collagen (UC-II) is known as an oral supplement, acting on joints by modulating the immune system response toward type II collagen, normally present at the cartilage level and altered during OA conditions^12^. As reported in experimental and clinical studies, UC-II supplementation is effective in decreasing cartilage degradation and improving the clinical condition^13–18^. The particular mechanism (oral tolerance) at the basis of the efficacy of UC-II is defined as the suppression of immune system reactivity toward type II collagen through the oral administration of undenatured antigen, involving the reciprocal interaction between dendritic cells and regulatory T cells^19,20^.

By using an untargeted approach, metabolomics encompasses the study of the most abundant small molecules (metabolites) in whole organisms, tissues, cells, biofluids, and culture media to better understand the metabolic changes occurring during physiological and pathological conditions^21–23^. This technique can be applied in a wide range of biological samples to obtain a chemical "fingerprint" originating from specific cellular processes that may be linked to different circumstances and may be useful for finding potential biomarkers for diagnostic and therapeutic interventions^24,25^. Among the analytical techniques used, ^1^H-Nuclear Magnetic Resonance spectroscopy (^1^H-NMR) has been extensively used in metabolomic studies in recent years because of its ability to observe and quantify simultaneously a wide number of structurally different metabolites, providing a wide range of information within a single experiment, without any component separation and with a minimal level of sample preparation and pretreatment^26,27^. ^1^H-NMR has been used widely in metabolomics studies for OA to investigate the osteoarthritic condition at the molecular level. ^1^H-NMR has proven to be extremely valuable in the assessment of different disease states and is also suitable as a potential diagnostic and prognostic tool^21,26,27^.

For the study of OA, particular interest was found in the analysis of different biological fluids, including urine, synovial fluid (SF), plasma, serum, and whole blood^24^. SF is the most common biofluid used in OA research due to its proximal localization to the pathological environment. Indeed, the degradation products, enzymes, and signal-transduction molecules implicated in OA are primarily released from the surrounding joint tissue into the SF, which could offer much biochemical information about the metabolic status of the affected joint^28–30^. In the literature, the metabolomic SF profile was investigated by ^1^H-NMR analysis in healthy and osteoarthritic joints of animals and humans^31–34^, identifying different metabolites associated with both conditions. Particularly, during OA, significant energy dysregulation, followed by increased lipid metabolism and altered glucose consumption, was observed^26^.

Very few metabolomic studies have been conducted in domestic animals, such as dogs and horses, and most of them have been carried out under experimental conditions or after traumatic OA^33,35–37^. These models are often related to an acute mechanical event with a consequent important inflammatory response. On the contrary, spontaneous OA is a more complex process which involve different factors including aging, obesity, metabolic conditions and activity. All these OA phenotypes are present in both human and canine spontaneous disease^38^. Thus, for the similar disease heterogeneity and progression, and for dog’s shortened lifetime and human-equivalent life stages, this specie has been indicated as a good model for human OA, particularly for the longitudinal assessment of the disease and its response to treatments^38^.

Currently, studies in which ^1^H-NMR analysis of SFs have been used to investigate the metabolic changes inside the joints of dogs suffering from spontaneous and supplemented with UC-II are missing.

In the authors’ opinion, the study of metabolomic joint profiles in a spontaneous canine model of OA might lead to the identification of a metabolic model with high clinical impact for the diagnosis and prognosis of the disease.

This study provides new insights into the pathological changes occurring in joint metabolism during OA and investigates the possible metabolic shifts induced by UC-II supplementation in dogs affected by the disease. For this reason, the first aim of the study was to compare the metabolomic profile of osteoarthritic dogs to that of healthy dogs to identify the discriminant metabolites associated with both scenarios. Our hypothesis was that ^1^H-NMR spectroscopy would be a valid analysis to create a predictive model that is able to differentiate between healthy and osteoarthritic dogs. The second aim of this study was to evaluate the clinical and metabolic effects of oral supplementation with UC-II for 30 days in dogs suffering from OA. Our hypothesis was that UC-II therapy would be effective in improving the joint condition and clinical signs, inducing metabolic changes detectable by the ^1^H-NMR technique.

## Results

### Experimental trial

**Figure 1:**
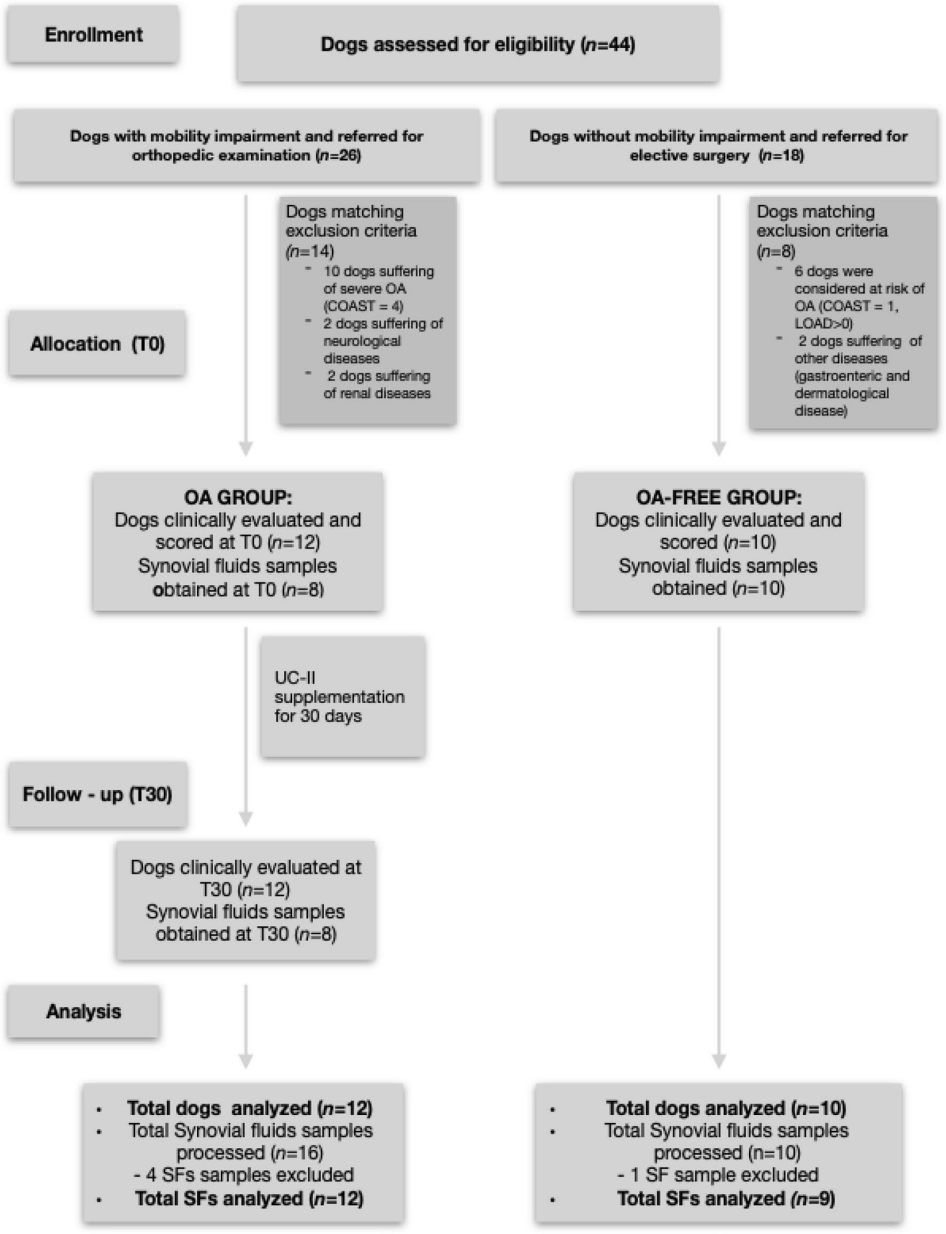
Flow diagram of the dog enrollment in the groups.

For the purpose of the study, 44 dogs that met the eligibility criteria were assessed. Particularly, among 26 dogs with mobility impairment and OA conditions screened, 14 were excluded because they met one or more of the exclusion criteria, and 12 were allocated to the OA group. Of 18 dogs without mobility problems, but referred for elective surgery, only 10 were allocated to the OA-free group after complete evaluation, while 8 were excluded because six of them were considered at risk of OA and the remaining two dogs were affected by concomitant diseases (Fig. 1). The demographic data for dogs enrolled in both groups are summarized in Table 1. In the OA group, the stage of the disease based on Canine OsteoArthritis Staging Tool (COAST) criteria was mild (stage 2) in 4 dogs and moderate (stage 3) in 8 dogs. In the OA group, for 8 dogs, we were able to obtain SF at the time of diagnosis (T0) and at the end of the 30-day treatment period (T30). In the OA-free group, for all dogs, we successfully collected SF samples from the stifle.

**Table 1:** Demographic data for each group. Data are expressed as mean and standard deviation (age and weight) or number.

	OA group	OA-free group
Dogs (n)	12	10
Age (months)	42 ± 36.9	24 ± 10
Weight (kg)	24.1 ± 8.49	26.5 ± 15.5
Sex (F/M)	6/6	4/6
Breed (n)	Beagle (3) German shepherd (3) Mixed breed (2) Labrador retriever (2) Pitt bull (1) Kurzhaar (1)	Mixed breed (4) Labrador (2) Pitbull (1) English setter (1) Caucasus mountain dog (1)
Joint examined (n)	Elbow (4) Hip (3) Shoulder (1)	Stifle (10)

### Clinical results for the OA group (dogs treated with UC-II)

**Figure 2:**
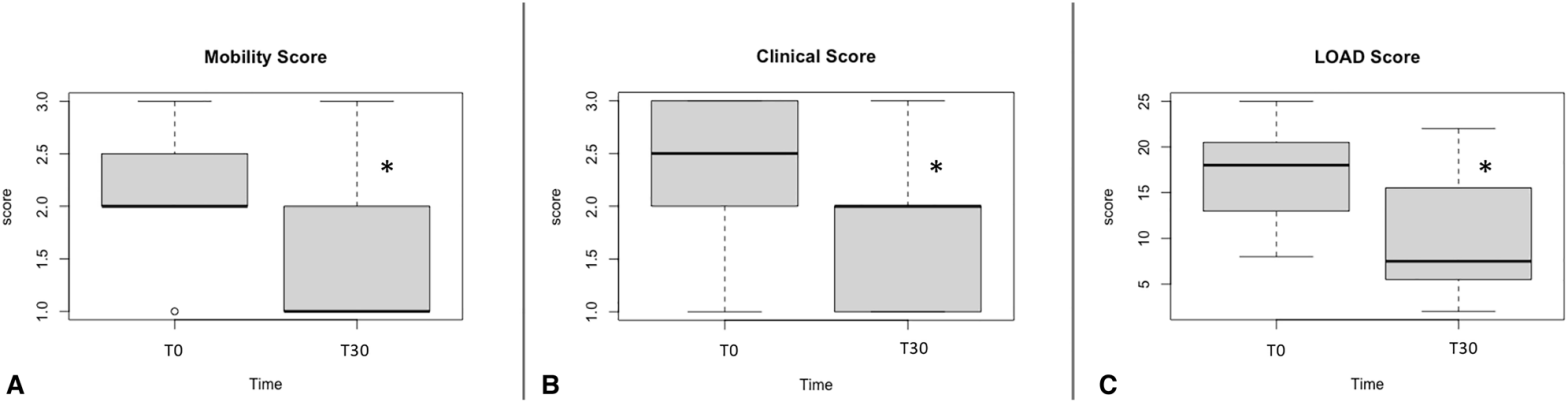
Boxplot for MOBILITY (**A**), CLINICAL (**B**) and LOAD (**C**) scores before (T0) and after (T30) UC-II supplementation. *P < 0.05 T30 compared to T0.

Based on veterinarian evaluation after UC-II supplementation (T30), the CLINICAL score (median and range) was lower at T30 [2 (1–3); P < 0.005] than at T0 [2.5 (1–3)]. Based on owner evaluations, the median Liverpool Osteoarthritis in Dogs (LOAD) questionnaire score was significantly reduced (P = 0.012) [7.50 (2–22)] at T30 when compared to T0 [18 (8–25)]; in addition, the MOBILITY score was lower (P = 0.04) at T30 [1 (1–3)] than at T0 [2 (1–3)] (Fig. 2A–C).

### ^1^H‑NMR characterization of-SF samples

**Figure 3:**
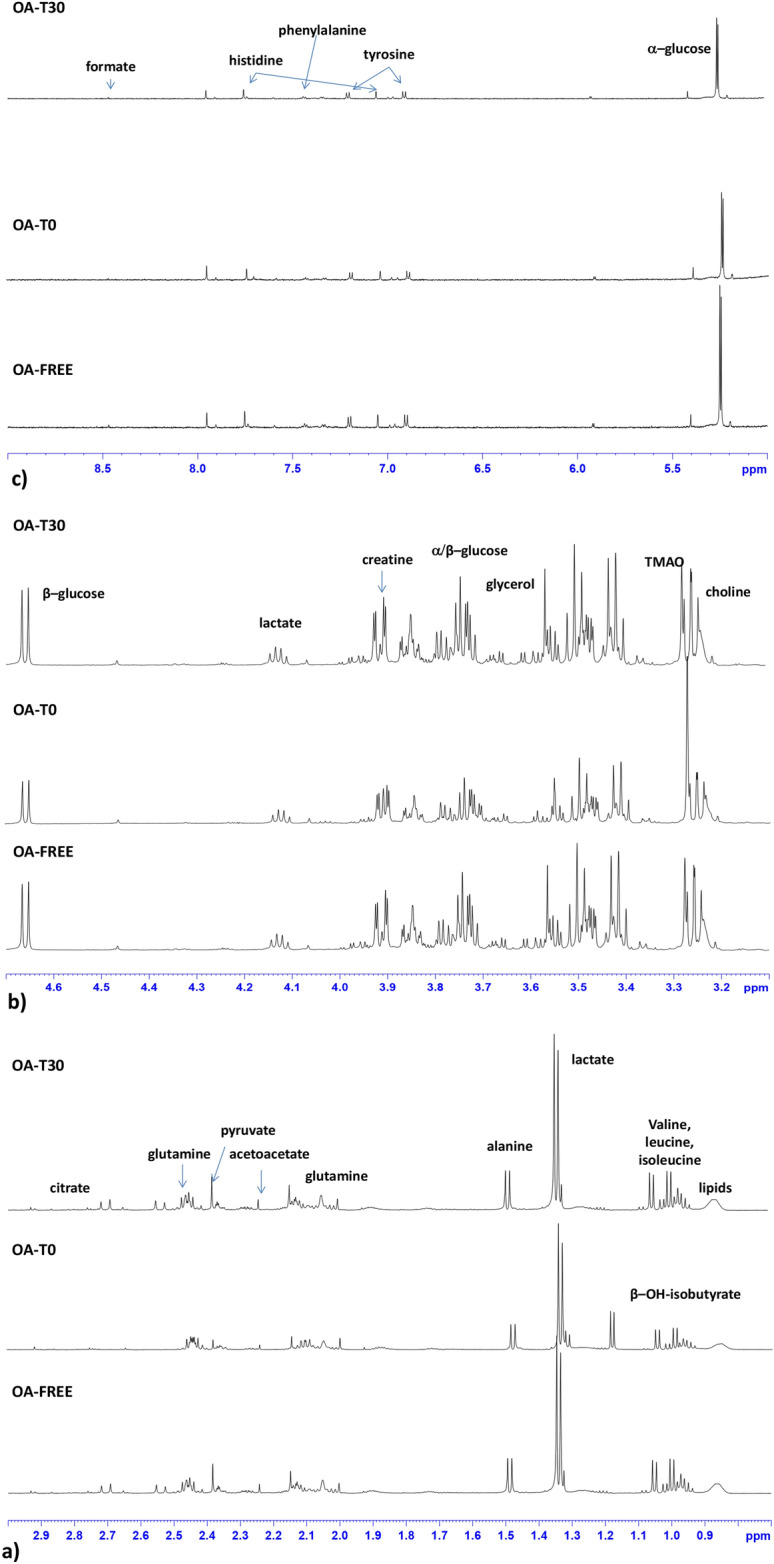
Stacked plot of 600 MHz typical cmpgpr ^1^H-NMR spectrum of OA-free, OA-T0 and OA-T30 SF samples. Expanded area in the range of (**a**) 0.5–3.00 ppm, (**b**) 3.00–5.00 ppm, and (**c**) 5.00–10 ppm. The assignment of the main peaks is indicated.

Representative 600 MHz ^1^H-NMR spectrum (1d cmpgpr) with identified metabolites for the canine SF samples from the OA group (OA-T0 and OA-T30) and OA-free group is shown in Fig. 3a–c. Visual inspection of the spectrum showed typical signals ascribable to lipids (0.9 ppm), isoleucine (0.99 ppm), leucine (1.01 ppm), valine (1.04 ppm), β-hydroxybutyrate (1.17 ppm), threonine (1.31 ppm), alanine (1.48 ppm), glutamine (2.10, 2.44 ppm), methionine (2.14 ppm), lactate (1.33 ppm), acetate (1.92 ppm), acetoacetate (2.23 ppm), pyruvate (2.37 ppm) and citrate (2.54 and 2.70 ppm) (Fig. 3a). Signals related to creatine/creatinine (3.04 and 3.90 ppm), β-glucose (3.25 and 4.65 ppm), trimethylamine-*N*-oxide (TMAO) (3.26 ppm) and lactate (4.12 ppm) were also observed (Fig. 3b). Then, in the aromatic region, histidine (7.06 and 7.90 ppm), phenylalanine (7.43 ppm), tyrosine (6.88 and 7.18 ppm) and formic acid (8.45 ppm) were identified (Fig. 3c).

### Multivariate statistical analysis

#### Unsupervised analysis

A preliminary unsupervised multivariate analysis (principal component analysis, PCA) (Supplementary Fig. S1) was performed on the bucket-reduced spectra for all of the studied samples with the aim of obtaining an overview of the data and revealing a possible data grouping of observations without any a priori-defined class. The obtained PCA model was described by 3 components (R2X = 0.723, Q2 = 0.465) with a good fit and classification of the model on the first principal component (t [1]), which explained 48.9% of the total variance. The scores plot showed the clustering of OA-free, OA-T0 and OA-T30 SFs. In particular, beside a marked separation between OA-free and OA-T0 samples, a clear dispersion of OA-T30 samples was also observed. These results could suggest a specific metabolic responses to the treatment for OA-T30. On the other hand, the compact clustering of OA-T0 samples indicates that the disease effects on the metabolic profiles prevail with respect to the expected variability within the considered cohort^39^. The HCA dendrograms (Supplementary Fig. S2) showed descriptive results similar to those of PCA: OA-T30 group samples are spread across two different clusters. The first exclusively contains OA-T30 samples and the second cluster also contains all OA-T0 samples. To refine the sample grouping observed in the unsupervised PCA model and to define the most reliable class-discriminating variables for the considered group controls, pairwise discriminant analyses were then performed.

### Supervised analysis and relative quantification of discriminant metabolites

**Figure 4:**
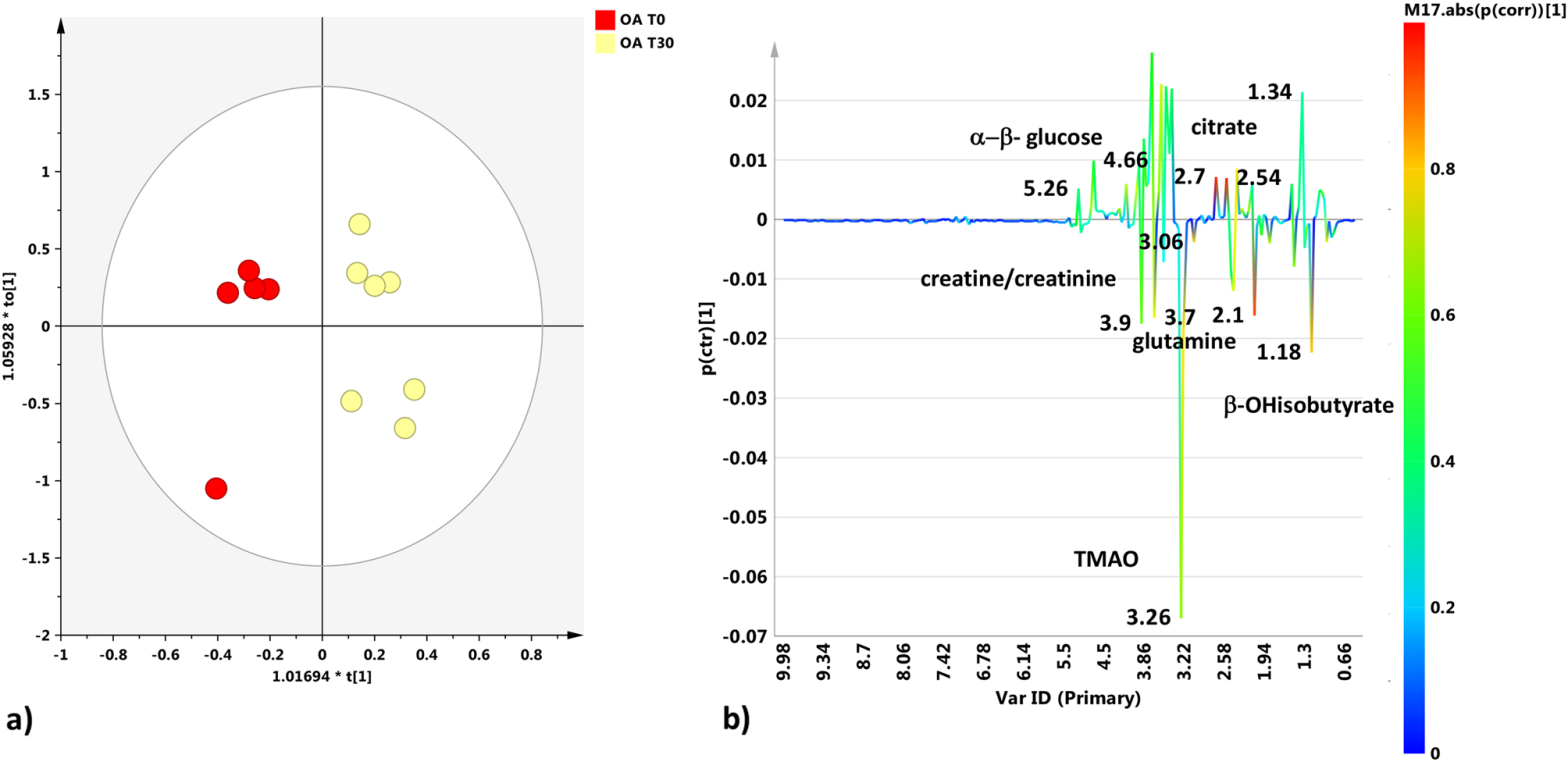
(**a**) OPLS-DA t[1]/t[2] scores plot for SF samples obtained at T0 and T30. (**b**) S line plot for the model colored according to the correlation-scaled coefficient (*p(corr) ≥|0.5|). The color bar associated with the plot indicates the correlation of the metabolites discriminating among classes.

**Figure 5:**
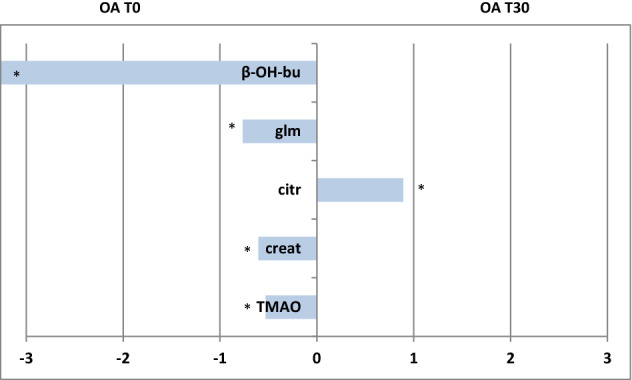
Discriminant metabolite comparison between OA-T0 and OA-T30 SF samples. The X-axis reports log2 fold change (FC) values. Metabolites with significant log2(FC) values are indicated with * (p value < 0.05) (*lip* lipids, *β-OH-bu* β-hydroxyisobutyrate, *Glm* glutamine, *cit* citrate, *creat* creatine, *TMAO* trimethylamine-*N*-oxide, *glu* glucose).

Orthogonal partial least squares discriminant analysis (OPLS-DA) also revealed a marked separation between the SF samples of OA-T0 and OA-T30. The obtained model was characterized by excellent descriptive and predictive parameters: one orthogonal and one predictive component gave R2X = 0.818; R2Y = 0.909; Q2 = 0.823 (Fig. 4a). By examining the S line plot of the molecular components discriminant for the two classes (Fig. 4b), OA-T0 samples showed higher contents of β-hydroxybutyrate, glutamine, TMAO and creatine/creatinine than OA-T30 samples. In contrast, OA-T30 samples showed a higher content of citrate than OA-T0 samples. The quantitative estimate of the discriminating power for the identified variables (selected buckets for specific metabolites signals) was described by the corresponding correlation parameter (pcorr) and VIP values (Supplementary Table S1). β-Hydroxybutyrate, glutamine, TMAO, creatine/creatinine and citrate exhibited a strong discriminating contribution to the model with high statistical reliability |pcorr|≥ 0.5 and strong discrimination power (VIP ≥ 1). The quantitative comparison between OA-T0 and OA-T30 samples for the SF discriminant metabolites, performed by considering the fold change (FC) ratio, is reported in Fig. 5.

**Figure 6:**
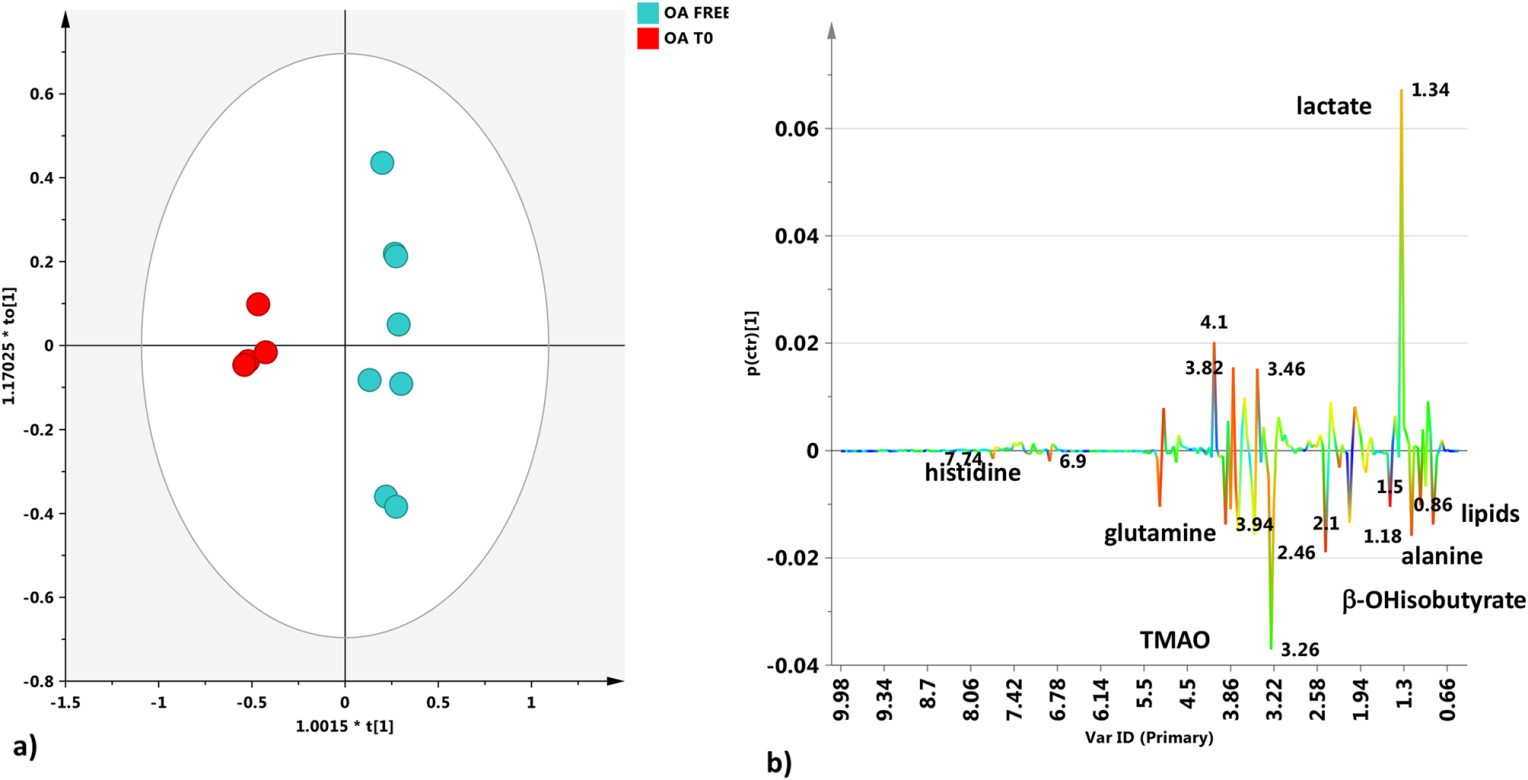
(**a**) OPLS-DA t[1]/t[2] scores plot for SF samples obtained from the OA-free and OA-T0 groups. (**b**) S line plot for the model colored according to the correlation-scaled coefficient (p(corr) ≥|0.5|). The color bar associated with the plot indicates the correlation of the metabolites discriminating among classes.

**Figure 7:**
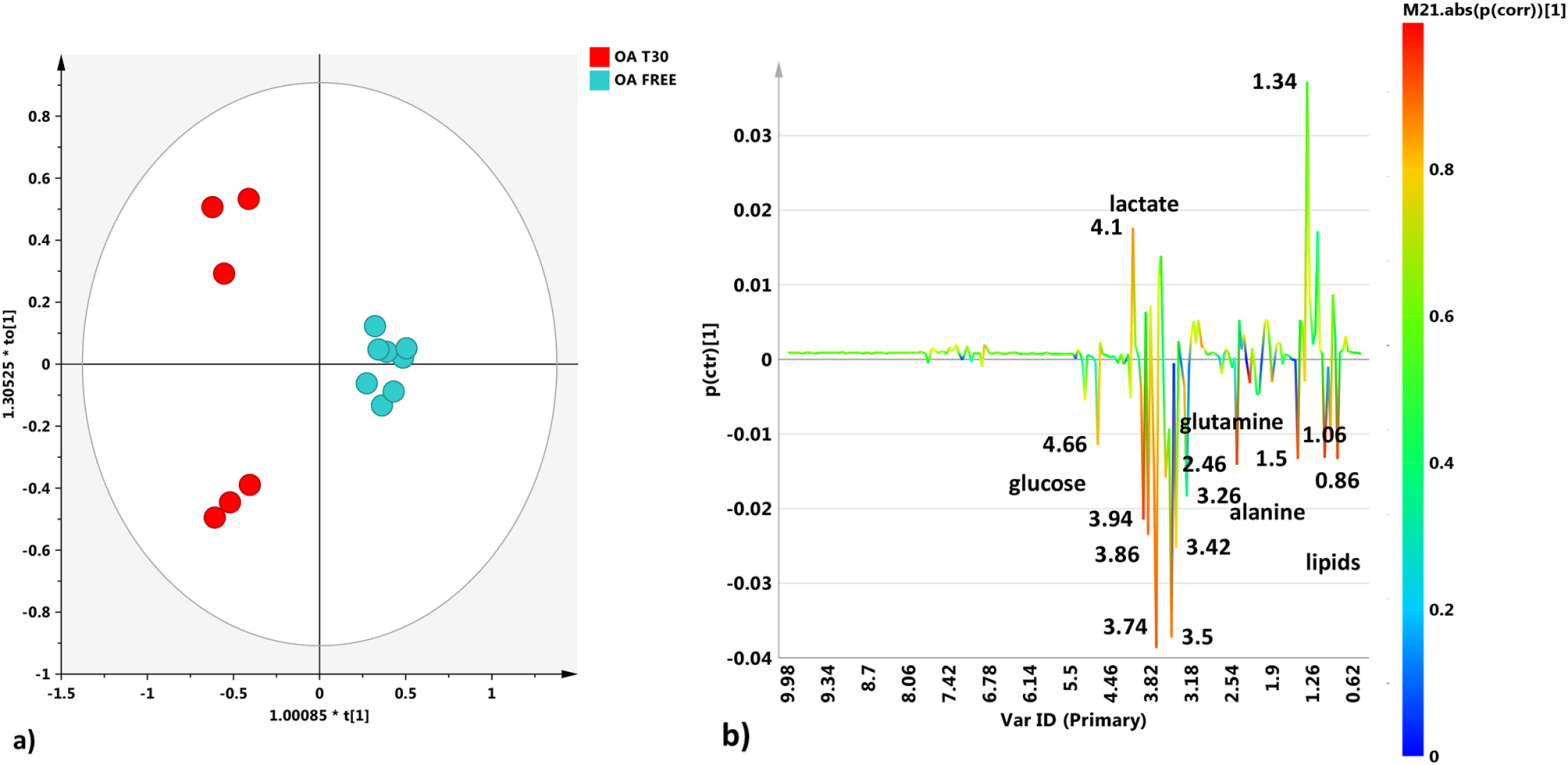
(**a**) OPLS-DA t[1]/t[2] scores plot for SF samples obtained from the OA-free and OA-T30 groups. (**b**) S line plot for the model colored according to the correlation-scaled coefficient (p(corr) ≥|0.5|). The color bar associated with the plot indicates the correlation of the metabolites discriminating among classes.

**Figure 8:**
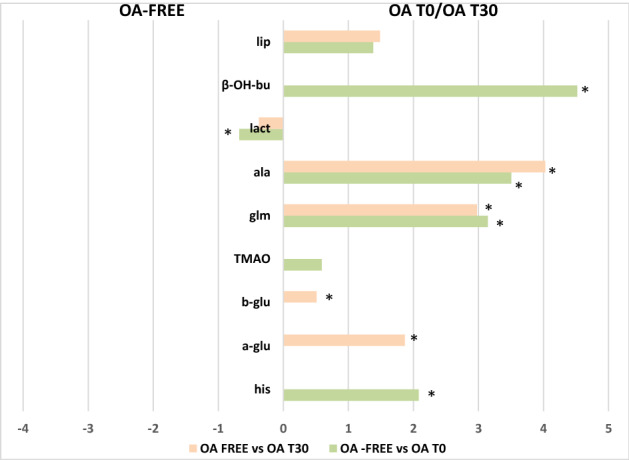
Discriminant metabolite comparison among OA-free, OA-T0 and OA-T30 SF samples. The X-axis reports log2 fold change (FC) values. Metabolites with significant log2(FC) values are indicated with *(p value < 0.05) (*lip* lipids, *β-OH-bu* β-hydroxyisobutyrate, *lact* lactate, *ala* alanine, *Glm* glutamine, *TMAO* trimethylamine-*N*-oxide, *glu* glucose, *his* histidine).

Multivariate statistical analysis was also performed by considering an OA-free group (control). In particular, a pairwise OPLS-DA analysis was performed by comparing the OA-free group with the OA-T0 group. The pairwise OPLS-DA analysis performed on OA-free and OA-T0 samples gave a good model (1 + 1 + 0 components gave R2X = 0.517, R2Y = 0.979 and Q2 = 0.903) (Fig. 6a). A clear separation between the two groups could be observed by visual inspection of the score plot for the model. The molecular components discriminating the two groups are reported in the corresponding S-line plot (Fig. 6b). Lipids, lactate, β-OH-butyrate, alanine, glutamine, TMAO and histidine were found to be discriminating metabolites with high statistical reliability and strong discrimination power (Supplementary Table S2). In particular, OA-T0 SF samples were characterized by higher lipid, glutamine, alanine, TMAO, β-OH-butyrate, and, in the aromatic region, histidine contents than OA-free SF samples. OA-free SFs showed a higher content of lactate than OA-T0 SFs (Fig. 6). The OPLS-DA pairwise analyses between OA-free and OA-T30 samples showed a clear separation between the groups and very good statistical parameters (one predictive and one orthogonal component, R2X = 0.651; R2Y = 0.969; Q2 = 0.912). The S line plot for the model revealed that the metabolites discriminating each group were lipids, glutamine, alanine and β-glucose, which showed higher levels in OA-T30 samples than in OA-free samples. For these metabolites, high statistical reliability and strong discrimination power were found (Supplementary Table S3). Moreover, in this pairwise analysis, OA-free SF was also characterized by a higher level of lactate than OA-T30 SF (Fig. 7). The identified discriminant metabolites of the OA-free SF samples versus OA-T0 and OA-T30 SF samples when compared considering the FC ratio (Fig. 8) showed a general higher content of lactate in OA-free samples than in OA SF samples. Specifically, SFs from the OA-T0 group revealed significant higher levels of β-hydroxyisobutyrate, alanine, glutamine and histidine with respect to the OA-free group. Samples from the OA-T30 group showed significantly higher contents of alanine, glutamine and α and β-glucose than samples from the OA-free group.

## Discussion

The results of this study demonstrate that the SF of the joints of dogs affected by OA has characteristic metabolic alterations compared to the SF of the joints of healthy dogs. Supplementation with UC-II for 30 days produced an improvement in clinical symptoms associated with mild to moderate OA, together with a characteristic modification in the SF metabolic profile. The clinical efficacy of UC-II found in this study confirms other results reported in the literature for dogs^14,15,17^.

Understanding how UC-II supplementation affects joint metabolism is important, given that metabolic reprogramming is a known feature of OA and this information will contribute to understanding the mechanism by which UC-II supplementation improves joint structure and function.

The metabolomic data found in this study suggest that ^1^H-NMR can be valid for the study, diagnosis and monitoring of the progression and treatment of OA^24^.

To our knowledge, this is the first study in which SF metabolomics have been applied to a spontaneous model of OA, which is a realistic translational model of the disease.

The discrimination comparison of the metabolic profiles obtained with ^1^H-NMR spectroscopy provides important new insights into the molecular pattern underlying OA disease and healthy conditions.

The observed elevation in β-hydroxybutyrate, found in OA-T0 samples (Figs. 3, 5), suggests that fat metabolism plays an important role as a source of energy in the osteoarthritic joint^5^. The elevation in β-hydroxybutyrate seems to correlate with the reduction in normal glucose metabolism. Indeed, evidence suggests that hydroxybutyrate and other ketone bodies are part of a regulatory mechanism affecting glucose and lipid metabolism in the osteoarthritic joint^35,36^. The absence of β-hydroxybutyrate in SF samples from the OA-free group confirms the characteristic role of this metabolite in the pathophysiology of OA (Figs. 3, 8). Similarly, the absence of β-hydroxybutyrate after UC-II supplementation (OA-T30 samples) was a surprising finding. This result suggests that this feed material is effective in metabolic rebalancing, resulting in an important reduction in lipid metabolism.

Glutamine showed a similar behavior to that of β-hydroxybutyrate (Figs. 4b, 5). Glutamine is an intermediate of the tricarboxylic acid cycle (TCA) and could be associated with altered oxidative metabolism^29^. The elevated glutamine could also be related to the decrease in glycosaminoglycan (GAG) production because glutamine is required for GAG production. In the literature, the majority of increased amino acid levels in OA SFs are considered to derive from hyaline cartilage collagen and proteoglycan destruction^5^. Thus, we could consider the glutamine finding observed here as an indicator of cartilage breakdown, correlated with the pathological condition^26,40,41^.

Among the other metabolites found to be elevated in OA-T0 samples, TMAO must be mentioned. TMAO comes from the conversion of choline and carnitine to trimethylamine *N*-oxide, and in various studies, TMAO has been reported to be a molecule associated with inflammation and the risk of metabolic disorders^42,43^. In a rheumatoid arthritis (RA) murine model, TMAO was proven to upregulate inflammatory pathways^44^ and drive these signaling events inside the joint^45^. In the present study, the finding of TMAO at high levels in OA SFs (especially reveled in OA-T0 samples) probably reflects its implication in the inflammatory state. It is not clear whether the presence of TMAO in SF could be associated with its production inside the joint or its transmigration from the serum to the articular region, promoted by the increased vessel permeability. Interestingly, creatine/creatinine levels were found to be significantly elevated in OA SF before treatment with UC-II. These metabolites have also been reported to be increased in human and animal studies performed on SF^5,27,46^. Creatinine is the product of creatine metabolism occurring during muscle breakdown. In this study, it is most likely that the observed increase in SF concentrations of creatinine is the result of the increased synovial membrane permeability consequent to inflammation, reflecting breakdown processes involving the muscles proximal to arthritic joints, proving that OA is a whole-joint disease that involves the surrounding soft tissue.

Furthermore, the results found in the metabolomic profile of OA SFs after UC-II supplementation are very intriguing, including the absence of β-hydroxybutyrate (mentioned before) and the higher concentration of citrate (Figs. 4b, 5). Citrate has been considered in the literature as a qualitative index of the Krebs cycle^5,35^.

In previous studies, similar results were found in osteoarthritic SFs and related to the increasing energetic demand promoted by fatty acid oxidation, which was considered sufficient to supply the acetyl-coenzyme A required for the Krebs cycle^35,36^.

Since the absence of β-hydroxybutyrate suggests a reduction in fatty acid metabolism, we can suppose that citrate could be derived from glycolysis and glucose metabolism. We also must consider that these samples came from osteoarthritic patients and, despite the UC-II supplementation, a higher energetic demand related to the pathological condition could be expected. In OA-T30 SFs, we also found high levels of glucose. The reports on glucose levels inside SF in the literature are mixed^41^. Some studies have reported an increased level of glucose^35^, while others have reported a reduction^47^. Animal studies report an increase in OA glucose concentration in equine (fivefold) and ovine (twofold) samples^33,48^.

Thus, the interpretation of glucose levels in different groups is difficult and confusing due to multiple influences, including consumption, altered glucose transport and synovial membrane permeability.

In addition, our results have shown some specific metabolites characterizing OA joints compared to healthy joints, independent of treatment. Alanine was found to be significantly elevated in OA SFs compared to healthy SFs, similar to glutamine as a marker of cartilage destruction. Furthermore, OA joints were characterized by higher levels of histidine than healthy joints. Similar results were found by Sitton et al.^49^, and the results are likely related to the demonstrated capability of OA chondrocytes to produce histamine under oxidative stress^35,50^.

The prevalence of aerobic or anaerobic environments in pathological or healthy joint conditions is an interesting consideration. Particularly, in our OA-free SF samples, elevated lactate was found (Figs. 6, 7, 8). Some studies link SF lactate elevation more to septic arthritis than to OA^31^. Other research groups have reported elevated lactate concentrations in OA SFs using different metabolomic techniques^51,52^. Overall, the role of lactate concentrations as a discriminant for OA seems very inconsistent, which is probably more due to the different analytic techniques used than the animal species evaluated and to the different stages of OA.

Thus, this untargeted metabolomic study does not attempt to precisely quantify all measurable metabolites in a sample, but provides their relative quantification according to the class samples differentiation (as fold change)^34,35,53^. Moreover, it should be considered that pulse programs that suppress unwanted compound signals are used in untargeted metabolomic NMR studies. The here used Carr–Purcell–Meiboom–Gill (CPMG) pulse sequence, which filters macromolecular signals, with water suppression, is the more practical and routinely applied for 1D NMR metabolomics to suppress resonances from macromolecular-derived signals^53^. This spectra acquisition technique, although very useful for quantitative metabolites comparison purposes, is not recommended for direct absolute quantification^54,55^.

This study has some limitations. First, the small number of dogs evaluated and the limited number of SFs obtained. The most common complication that allowed the loss of patients was the difficulty in obtaining a sufficient volume of SF sample by arthrocentesis. It has been proven that in certain disease states, especially in more compromised OA joints, a negative tap can be obtained. Second, age was unbalanced among groups. Indeed, the OA-free group was exclusively characterized by healthy young dogs. Therefore, the results found for this group of dogs may differ from those of other healthy older dogs without OA. Furthermore, the choice to not perform the second (T30) SF sampling in the OA-free group could be considered a methodological bias in terms of capturing possible changes in the metabolomic status of the same healthy joints after one month. Obviously, this decision was related to the clinical nature of the study and ethical concerns.

Another potential limitation of the study should be the different breeds analysed in the OA group and OA-free group. Indeed, it is possible that distinct breeds could potential have different metabolomic profiles that may be responsible for the differences observed. Additionally, for the feasibility of the study, we considered different joints for OA group and OA-free group. In the OA group the joint of interest was the one with the most severe clinical and radiographic signs of OA. In the OA free group, knee was chosen arbitrary considering feasibility and safety of SF sampling at this joint. From this point of view, we may not exclude that distinct metabolomic profiles may be linked to joint-specific environment and pathophysiological pathways.

Clinical improvements recorded in this study were based on the veterinarian and owner evaluation. Although the scoring systems used in this study has been validated and already used in previous clinical studies^17,56–59^, the use of an objective tool, as gait analysis mat, for kinematic and kinetic evaluation, might have been desirable for the aim of this study, but unfortunately not applicable at time of the data collection.

Concluding, this is the first study in which HNMR analysis has been used to investigate the synovial fluid metabolism during spontaneous OA condition in dogs supplemented with UC-II and compared to healthy control. The analysis of SFs showed a marked separation for OA-free and OA-T0 samples, a clear dispersion of OA-T30 samples. These results could suggest a specific metabolic response to the treatment for OA-T30, confirmed also by the clinical scores’ improvement.

Considering the cited limitations, we would underline the preliminary nature of the study. More advanced and extensive clinical studies using UC-II have been initiated by our research group, which include metabolomic analysis and more objective clinical evaluation (gait analysis).

## Materials and methods

### Study design

The study was designed as a clinical controlled, randomized and prospective study, performed after the approval of the ethical committee of Veterinary Clinical and Zootechnical Studies of the Department of Emergency and Organs Transplantations (DETO/223/III/13/2018). All methods were performed in accordance with the Italian guidelines and regulations and the study has been reported in accordance with the ARRIVE guidelines. For the purposes of this study, client-owned dogs with mobility impairment, evaluated at the Surgical Unit of the Section of Veterinary Clinics and Animal Production of the Department of Emergency and Organ Transplantation of the University of Bari, were screened. Moreover, dogs without mobility impairment and with no clinical signs of orthopedic diseases, referred for elective neutering surgery, were also screened to recruit dogs with healthy joints for enrollment in the control (OA-free) group. For each dog involved in the study, informed consent was signed by the owner at the time of enrollment. All dogs underwent preliminary complete physical, clinical chemical and hematological evaluations.

The inclusion criteria for the OA group were the contemporary observation of the following criteria: a history of mobility impairment; the presence of lameness, pain and radiographic signs of OA (articular incongruence, subchondral sclerosis, and the presence of osteophytes and bone deformation) in at least one appendicular joint; at least a 3-week washout period from any previous anti-inflammatory/analgesic/nutraceutical therapy; and a score of 2 or 3 on the COAST^56^. The exclusion criteria for OA dogs were the observation of at least one of the following: the diagnosis of a neurological disease, the presence of a comorbidity such as major cardiovascular and respiratory dysfunctions and other chronic affections (dental, renal, or hepatic diseases), and no signs of OA evident in the radiographic examination.

The inclusion criteria for the dogs in the OA-free group were the absence of any sign of mobility impairment and joint disease based on the history, an orthopedic examination and a score equal to 0 on the LOAD^59^ questionnaire and the COAST.

### Orthopedic examination and staging of OA

At the beginning of the clinical evaluation, a complete history was collected from the owners, including any episode of trauma, the evolution of the lameness and the features of the mobility impairment observed at home.

The severity (stage) of OA was assessed based on COAST criteria, considering the impact of the disease on the whole dog (the grade of the dog) and on the joint(s) (the grade of the joint). The use of this tool allowed us to integrate the information obtained from the orthopedic evaluation with the information provided by the owner (Supplementary material—Table S4)^56^.

Orthopedic examination was performed for all dogs by the same experienced clinician. The examination included an evaluation of posture, a gait analysis, and the presence of articular pain and range of motion (ROM) at joint manipulation following the criteria described in the supplementary material (Supplementary Table S4). Once the painful joint(s) were identified, a radiographic examination was performed to confirm the diagnosis, and a score was assigned based on the severity (Supplementary Table S4).

Owners were asked to complete the LOAD survey^59^, and based on the final score obtained, each patient was assigned to a specific grade of mobility alteration (MOBILITY score, from 1 to 4).

The owners were also asked to grade the degree of discomfort of the animal, which was scored from 1 to 4 (Supplementary Table S4).

Considering all scores recorded, the final COAST stage was achieved by choosing the highest score between the “grade of the dog” and the “grade of the joint”. Thus, the final OA stage of dogs was scored as follows: 0, clinically normal, with no risk factors for developing OA; 1, clinically normal, with risk factors for developing OA; 2, mild; 3, moderate; and 4, severe. Dogs with a COAST score of 2 or 3 were included in the OA group, while only dogs with a COAST score of 0 were included in the control group.

All dogs in the OA group were treated with UC-II, one tablet (40 mg) daily, for 30 days from the time of diagnosis (T0). At the end of the treatment period (T30), all dogs were re-evaluated as previously described.

### SF sampling

To investigate the metabolomic profile by ^1^H-NMR analysis, all dogs included in the study underwent SF sampling. For the OA group, sampling was performed at the beginning (T0) and after 30 days (T30) of treatment with UC-II considering the most affected joint. Arthrocentesis was performed after X-ray examination, with patients heavily sedated or under general anesthesia. The joint to be sampled was clipped and aseptically prepared. The procedure was always performed by the same operator. For sampling, needles of appropriate length were chosen related to the size of the patient and the anatomy of the interested joint. Each sample collected was stored in a sterile Eppendorf tube at a temperature of − 20 °C until the ^1^H-NMR measurements.

In the OA-free group, the stifle joint was chosen as the elective joint for the evaluation of SF required for the purpose of the present study. SF collection was performed from one stifle joint during anesthesia at the end of the surgical procedure. To confirm the absence of signs of OA in these dogs, the X-ray study of the joint was performed at the time of preanesthetic sedation.

### SF sample preparation for NMR analysis

A total of 16 and 10 samples of SF were collected and processed for ^1^H-NMR analysis from the OA group and OA-free group, respectively. The analysis of SF samples was performed at the General and Inorganic Chemistry Laboratory of Department of Biological and Environmental Sciences and Technologies (DiSTeBA), University of Salento. For each sample, a progressive identification number was assigned. Samples were spun down in a microcentrifuge at 14,000 rpm for 15 min (temperature 4 °C). Then, 420 μL of each SF sample was added to 280 μL of D_2_O containing 0.05% w/v TSP-*d*4 (sodium salt of trimethylsilyl propionic acid) as a chemical shift reference, δ = 0 ppm, and filled in a 5 mm NMR tube. At least 500 μL of SF was collected to consider the sample valid for the analysis. Four samples from the OA group and one sample from the OA-free group were excluded from statistical analysis because they showed the typical signals ascribable to ethanol, probably related to the antiseptic scrubs performed during the collection procedure. The presence of the ethanol signal (1.18 ppm) causes the failure to identify the typical signal of β-hydroxybutyrate (1.17 ppm).

### ^1^H-NMR spectra acquisition and processing

All measurements were performed at 300 K on a Bruker Avance III 600 Ascend NMR spectrometer (Bruker, Karlsruhe, Germany) operating at 600.13 MHz, equipped with a TCI CryoProbe (inverse triple resonance Cryoprobe Prodigy), and incorporating a z-axis gradient coil and automatic tuning-matching. Experiments were acquired in automation mode after loading individual samples on an integrated Bruker Automatic Sample Changer interfaced with IconNMR software (Bruker). For each sample, a one-dimensional ^1^H-NMR spectrum with a Carr–Purcell–Meiboom–Gill spin-echo sequence (CMPG) filter to attenuate signals from macromolecules was acquired by using a standard Bruker pulse sequence (cpmgpr 1d) in a spectral window of 20.0276 ppm (12,019.230 Hz), with 32 scans and 90 10.94-ms pulses. The identification and assignment of the metabolites was determined by the analysis of two-dimensional homo- and heteronuclear NMR spectra (2D 1H J-resolved, ^1^H COSY, ^1^H–^13^C, HSQC, and HMBC) and by comparison with the literature data^33,35–37^.

### Statistical and multivariate analysis

Demographic and clinical data were analyzed with R Statistic software^60^. Normal distribution was evaluated with the Shapiro test, and the median and range were calculated for each parameter. The Wilcoxon signed rank test for two dependent samples was used to capture significant variations in LOAD, MOBILITY and CLINICAL scores between T0 and T30 in the OA group.

The ^1^H-NMR spectra were processed using Topspin 3.6.1 and Amix 3.9.13 (Bruker, Biospin, Italy), both for simultaneous visual inspection and the successive bucketing process. All NMR spectra (in the range 10.0–0.9 ppm) were segmented in fixed rectangular buckets of 0.04 ppm width (normal rectangular bucketing) and successively integrated. The bucket tables thus obtained were subjected to a standardization procedure to minimize the possible differences in the concentration of the various metabolites due to sample preparation and/or acquisition conditions. The spectral region between 5.10 and 4.7 ppm was discarded because of the residual peak of water. Total sum normalization, by which the bucket integrals are divided by the total spectral intensity, was applied to minimize small differences due to sample concentration and/or experimental conditions among samples^61^. The data set (bucket table) resulted in a matrix corresponding to the bucketed ^1^H-NMR spectrum values (in columns) measured for each sample (in rows). The Pareto scaling procedure was applied, performed by dividing the mean-centered data by the square root of the standard deviation^61^. After the data processing step, multivariate statistical analysis (unsupervised PCA, Hierarchical Clustering Analysis (HCA) and supervised OPLS-DA) was performed to examine the intrinsic variation in the ^1^H-NMR data using SIMCA 14 software (Sartorius Stedim Biotech, Umeå, Sweden). Unsupervised PCA was aimed at extracting the maximum possible information from a multivariate data structure^62^. HCA was performed as complimentary data reduction and pattern recognition method on the data and the resulting dendrogram was calculated using the Ward distance algorithm^63^. The robustness of the statistical models was tested by the cross-validation default method (sevenfold) and further evaluated with a permutation test (20 permutations) available in SIMCA-P software^62^. The quality of the models was described by R2 and Q2 parameters. The first (R2) is a cross-validation parameter that describes the goodness of fit. The second (Q2) represents the portion of variance in the data predicted by the model. Cross-validated analysis of variance (p[CV − ANOVA]) provides a p value indicating the level of significance of group separation in OPLS-DA^64,65^. The variables responsible for the observed discrimination were identified by using the statistical tool S line plot. The quantitative estimate of the discriminating power for the variables was described by the corresponding weight (wc*) and the correlation parameter (pcorr) values. More quantitative estimates of the discriminatory power for each of the variables were described by the VIP values parameter^62^. The relative change in discriminating metabolite content between the observed groups was evaluated by analyzing the mean values ± standard deviation of selected bucket-reduced distinctive unbiased NMR signals after spectra normalization. In particular, the changes in metabolite levels between two groups were calculated as the log2-FC ratio of the normalized median intensity of the corresponding signals in the spectra of the groups^66,67^. Statistical significance was set at least at an adjusted p value < 0.05.

## Supplementary Information

Supplementary Information 1.

Supplementary Information 2.

## Data Availability

The datasets used and/or analysed during the current study available from the corresponding author on reasonable request.
