# Supplementary material for: 1H-NMR metabolomic profile of healthy and osteoarthritic canine synovial fluid before and after UC-II supplementation
Source: Sci Rep. 2022 Nov 16;12:19716. doi: 10.1038/s41598-022-23977-1 (PMC9669020; doi:10.1038/s41598-022-23977-1)
Supplement: Supplementary file 1 — Supplementary Information 1. [file 41598_2022_23977_MOESM1_ESM.pdf]

^{1}H-NMR metabolomic profile of healthy and osteoarthritic canine synovial fluid before and after UC-II supplementation

Marzia Stabile[1,*,+], Chiara Roberta Girelli[2,+], Luca Lacitignola[1], Rossella Samarelli[3], Antonio Crovace[1], Francesco Paolo Fanizzi[2], and Francesco Staffieri[1]

[1]Section of Veterinary Clinics and Animal Production, Department of Emergency and Organ Transplantation, University of Bari, 70123, Bari, Italy
[2]Department of Biological and Environmental Sciences and Technologies, University of Salento, 73100 Lecce, Italy
[3]Section of Avian Pathology, Department of Veterinary Medicine, University of Bari, 70123, Bari, Italy

[*] marzia.stabile@uniba.it ; Francesco.staffieri@uniba.it

[+]these authors contributed equally to this work

Supplementary information

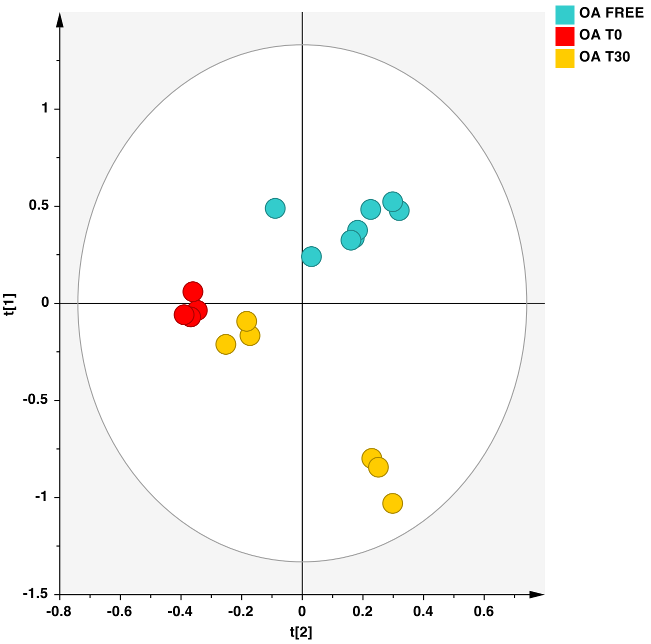

Figure S1. PCA t[1]/t[2] scores plot of the whole SF samples data set. Three components give R2X=0.731; Q2= 0.453

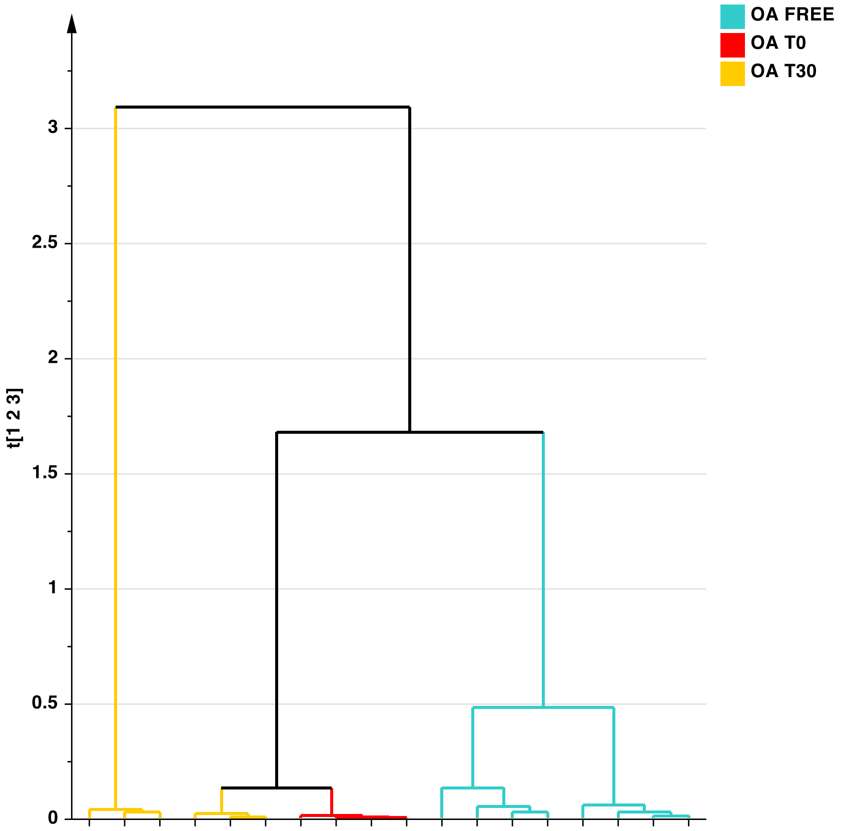

Figure S2. HCA dendrogram showing the clusters generated by hierarchical cluster analysis of the whole data set using Ward linkage criterion. Dendrograms are sorted by size.

**Var ID (Primary)**	**M17.p(corr)[1]**	**M17.VIP[1+1+0]**
**creatine/creatinine (3.9 ppm)**	-0.598987	2.55625
**TMAO (3.26 ppm)**	-0.634953	5.13238
**citrate (2.7ppm)**	0.961914	1.83663
**citrate (2.54 ppm)**	0.961048	1.82662
**glutamine (2.1 ppm)**	-0.920607	2.73875
**β-OH-butyrate (1.18 ppm)**	-0.795191	3.02273

Table S1 List of discriminating chemical descriptors (variables) with corresponding correlation coefficient (pcorr), and variable importance on the projection (VIP) for OA-T0 and OA-T30 SF samples OPLS-DA model

**Var ID (Primary)**	M22.p(corr)[1]	M22.VIP[1+1+0]
**histidine (7.74 ppm)**	-0.870006	0.816922
**TMAO (3.26 ppm)**	-0.63912	4.43003
**glutamine (2.1 ppm)**	-0.835927	2.67689
**alanine (1.5 ppm)**	-0.985323	2.41377
**β-OH-butyrate (1.18 ppm)**	-0.721501	2.15177
**lactate (1.34 ppm)**	0.616009	5.44766
**lipids (0.86 ppm)**	-0.95764	2.76259

Table S2 List of discriminating chemical descriptors (variables) with corresponding correlation coefficient (pcorr), and variable importance on the projection (VIP) for OA-FREE and OA- T0 SF samples OPLS-DA model

**Var ID (Primary)**	**M37.p(corr)[1]**	**M37.VIP[1+1+0]**
**α-glucose (5.26 ppm)**	-0.923619	2.29777
**β-glucose (4.66 ppm)**	-0.925983	2.10316
**glutamine (2.1 ppm)**	-0.94806	2.33689
**alanine (1.5 ppm)**	-0.917508	2.24029
**lactate (1.34 ppm)**	0.548916	2.96059
**lipids (0.86 ppm)**	-0.931488	2.26821

Table S3 List of discriminating chemical descriptors (variables) with corresponding correlation coefficient (pcorr), and variable importance on the projection (VIP) for OA-FREE and OA- T30 SF samples OPLS-DA model
