# Supplementary material for: 1H-NMR metabolomic profile of healthy and osteoarthritic canine synovial fluid before and after UC-II supplementation
Source: Sci Rep. 2022 Nov 16;12:19716. doi: 10.1038/s41598-022-23977-1 (PMC9669020; doi:10.1038/s41598-022-23977-1)
Supplement: Supplementary file 2 — Supplementary Information 2. [file 41598_2022_23977_MOESM2_ESM.pdf]

Supplementary information

Orthopedic Scoring System				
	**1**	**2**	**3**	**4**
**Static posture**	normal	mildly abnormal: subtle abnormality of limb loading, subtle shifting in static body weight distribution	moderately abnormal: obvious abnormality in limb loading, obvious shift in static body weight distribution	severely abnormal: restless when standing, reluctance (difficulty) to stay standing, severe shift in static body weight distribution, severely abnormal limb loading
**Gait**	normal: symmetry, appropriate weight bearing, appropriate body weight distribution, fluent gait	mildly abnormal: possibly affected at some gaits or with some activities, subtle stiffness in gait, subtle changes in body weight distribution, subtle asymmetry, subtle lameness, no difficulty rising (getting up)	moderately abnormal: consistent abnormalities in motion at all gaits and activities, obvious stiffness in gait, obvious changes in body weight distribution, obvious reduction in use of affected limb, obvious decrease in stance phase, some difficulty rising	severely abnormal: struggles to move/ reluctant to move, severe lameness usually present, severe weight shift, marked difficulty rising.
**Range of motion**	normal: full ROM of the joint, no presence of instability, no presence of crepitation	mildly abnormal: subtle alteration, no presence of instability, no presence of crepitation	moderately abnormal: obvious alteration, possible presence of slight instability and crepitation	severely abnormal: severe alteration, presence of instability of the joint, presence of crepitation and click sounds.
**Pain of Joint**	none: no signs of pain or discomfort during manipulation	mild: subtle signs of pain and discomfort during the manipulation	moderate: evident signs of pain during manipulation	severe: severe signs of pain during manipulation
**X- Ray**	no signs	mild: evidence of articular incongruence, possible subchondral sclerosis, absence of osteophytes	moderate: evidence of articular incongruence, evident subchondral sclerosis, scarce osteophytes	severe: evidence of articular incongruence, evidence of subchondral sclerosis, presence of osteophytes, bone deformations.

Table S4. The score used for each parameter assessed during the orthopedic examination adapted from the COAST criteria of Cachon *et al*., 2018.
